# Absence of West Nile and Usutu Virus Persistence in Overwintering Mosquitoes in Northeastern France: Insights from Cold-Season Surveillance

**DOI:** 10.3390/v17091217

**Published:** 2025-09-06

**Authors:** Pauline Jourdan, Jean-Philippe Martinet, Hubert Ferté, Bruno Mathieu, Marie Vazeille, Jérôme Depaquit, Anna-Bella Failloux, Anouk Decors, Rémi Charrel

**Affiliations:** 1 Unité des Virus Émergents (UVE: Aix-Marseille Univ, Università di Corsica, IRD 190, Inserm 1207, IRBA), 13005 Marseille Cedex 05, France; pauline.jourdan-sessarego@etu.univ-amu.fr; 2 Faculté de Pharmacie, Université de Reims Champagne Ardenne, UR ESCAPE-USC ANSES PETARD, 51 rue Cognacq-Jay, 51096 Reims Cedex, France; 3 Instituts de Bactériologie et Parasitologie, Faculté de Médecine, Maïeutique et Sciences de la Santé, Université de Strasbourg, UR 3073-PHAVI, 3 rue Koeberlé, 67000 Strasbourg, France; 4 Institut Pasteur, Université Paris Cité, Arboviruses and Insect Vectors, 75015 Paris, France; 5 Office Français de la Biodiversité, Direction de la Recherche et de l’Appui Scientifique, 45071 Orléans Cedex, France

**Keywords:** overwintering mosquitoes, West Nile virus, Usutu virus, northeastern France, *Culex pipiens*, arthropod-borne, bird, diagnosis

## Abstract

Emerging arboviruses of the *Orthoflavivirus* genus such as West Nile virus (WNV) and Usutu virus (USUV), primarily transmitted by *Culex* mosquitoes, pose significant public health threats due to their ability to cause severe neurological diseases in humans and animals. While studies in North America and Central Europe have shown that these viruses can persist in overwintering mosquitoes, their role in viral maintenance during the cold season in northeastern France remains unknown. This study aimed to assess whether overwintering female mosquitoes in this region could harbor WNV or USUV during the cold season, potentially maintaining viral circulation until the following transmission season. Between October 2021 and February 2024, a total of 10,617 overwintering female mosquitoes were collected in various types of habitats across five departments in northeastern France. The most common species was *Culex pipiens* (88%). Mosquitoes were grouped into 1121 pools (1–10 individuals each) and tested by real-time RT-PCR for WNV, USUV, and other flaviviruses using a pan-Flavivirus NS5-targeting assay. All pools tested negative, indicating no evidence of viral RNA in overwintering females. These results suggested that overwintering female mosquitoes in northeastern France do not act as reservoirs for WNV or USUV, and do not contribute to their overwintering maintenance.

## 1. Introduction

West Nile (WNV) and Usutu (USUV) viruses are two emerging arboviruses belonging to the *Orthoflavivirus* genus. They are mainly transmitted through the bites of infected *Culex* spp. mosquitoes [1]. Other modes of transmission have also been described in the literature, but they are less common [2,3,4]. These viruses are maintained in an enzootic cycle involving passeriform (mainly common blackbird *Turdus merula*) and strigiform birds as reservoirs on one hand and ornithophilic mosquitoes, particularly *Culex pipiens* on the other hand. These mosquitoes are endemic to France, and their activity period extends from June to late November, meaning that most WNV or USUV cases occur during the summer or fall seasons. These viruses can also be transmitted to dead-end hosts [5], including [6]: horses [7,8,9,10], dogs [7,8], rodents [11], squirrels [12], wild boar [13,14] and roe deer [14]. The virus has also been detected in bats, whose epidemiological role is as yet undetermined [15]. In humans, a wide range of symptoms are described in the literature, from flu-like illness to severe neurological disorders as encephalitis or meningitis [16]. For several years, WNV has been responsible for multiple outbreaks, the most well-known being the 1999 outbreak in New York. Following this outbreak, a study was conducted to determine whether WNV could survive through the winter by being maintained in overwintering female mosquitoes. In New York City, researchers tested and found multiple pools of overwintering *Culex* mosquitoes positive for WNV RNA [17]. Another study conducted in California showed that several factors contributed to WNV persistence during the cold season [18]. WNV can be vertically transmitted in *Aedes* mosquitoes, allowing the virus to be passed to the offspring of overwintering mosquitoes in laboratory experiments [19]. Virus is transmitted in vivo to the eggs of an infected mosquito [20].

In France, avian mortality data from 2018 suggested the endemization of USUV, particularly in certain regions such as Centre-Val de Loire, Ile-de-France and Pays de la Loire [21]. Mosquitoes were captured around bird outbreak areas that emerged during the project to assess their transmission potential. The French network for the epidemiological surveillance of wildlife diseases (SAGIR) operates surveillance through the collection of dead or moribund birds and mammals (opportunistic sampling method). This participatory surveillance program aims at detecting as early as possible abnormal mortality or morbidity signals. A total of 33 outbreaks across metropolitan France (representing 47 bird carcasses) were analyzed and recorded in the database from 2020 to 2022. After the 2018 epizootic in blackbird populations with high mortality [22], there was little mortality afterwards until 2022 in mainland France. Since 2022, it appears that the mortality had increased [23].

As part of the surveillance of WNV and USUV, several mosquito collection campaigns were conducted between 2021 and 2024 in northeastern French departments where infected birds had been identified. These collections aimed to examine mosquito species potentially involved in the overwintering persistence of these viruses.

The aim of our study was therefore to estimate the transmission of two arboviruses, West Nile and Usutu viruses, by overwintering mosquitoes in northern France and to determine whether overwintering females play a role in maintaining these viruses during the cold season within the natural virus cycle.

## 2. Materials and Methods

### 2.1. Mosquitoes Field Collections

**Figure 1:**
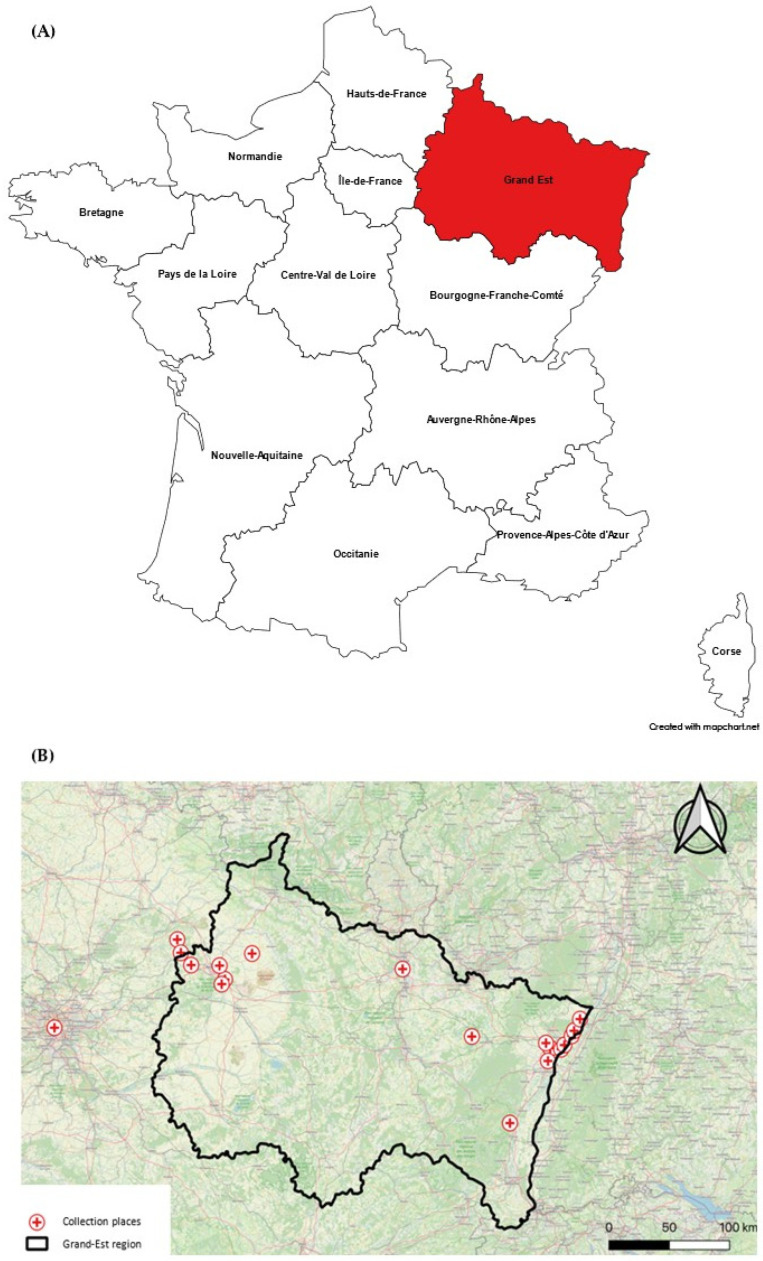
(**A**) French regions maps, the Grand-Est region is highlighted in red; (**B**) trapping sites in the Grand-Est region.

**Figure 2:**
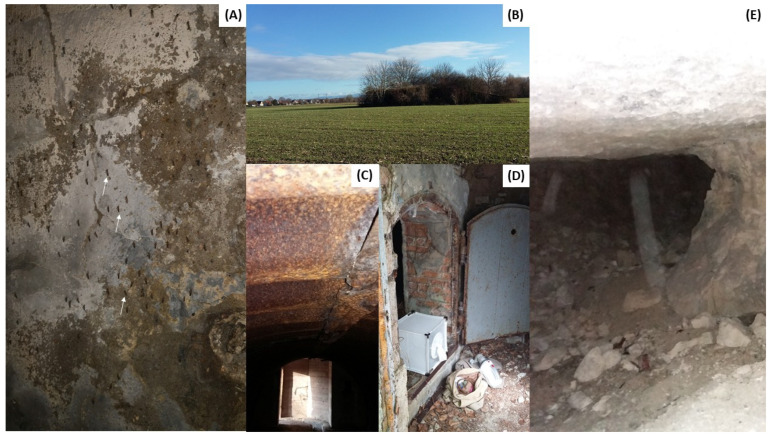
Images of different mosquito collection sites. (**A**) Offendorf (white rows show overwintering mosquitoes); (**B**) Gambsheim (hunting ground); (**C**) Faux-de-Verzy (World War I bunker); (**D**) Mulhouse Zoo (the bear enclosure); and (**E**) Glennes (World War 1 tunnel).

Between October 2021 and February 2024, five mosquito field surveys were conducted in five departments of northeastern France: Moselle, Aisne, Marne, Haut-Rhin, and Bas-Rhin where dead birds infected with USUV were found [24] (Figure 1). A total of 10,617 overwintering female mosquitoes were collected during these surveys. An insect pooter was used to collect mosquitoes in caves, bunkers, and hunting grounds, which were known to serve as overwintering habitats [25] (Figure 2).

Various mosquito species were collected from these different locations: *Culex pipiens*, *Culiseta annulata*, *Anopheles maculipennis*, *Anopheles plumbeus*, *Culex territans*, *Culex hortensis*, and *Aedes vexans*.

When overwintering female mosquitoes were captured, specimens of the same species were grouped into pools of up to ten individuals. The number of mosquitoes in each tube ranged from 1 to 10.

### 2.2. Total Nucleic Acid Purification

Female mosquito pools were homogenized in 1000 µL of Minimum Elementary Medium (MEM) supplemented with 3% of Amphotericin B, 1% of Kanamycin, 1% of Penicillin-Streptomycin as previously described [26].

A 100 µL aliquot of the homogenized mosquito sample was mixed with 100 µL of PBS buffer and 100 µL of lysis buffer. Viral RNA was extracted using the QIAamp Viral RNA Mini Kit (Qiagen, Hilden, Germany), following the manufacturer’s instructions. The final elution volume was 90 µL. All collected female specimens were tested for WNV and USUV RNA detection.

### 2.3. WNV and USUV RNA Detection

A combination of primers was used with two WNV-specific (WNV duo) and two USUV-specific (USUV duo) real-time reverse transcription polymerase chain reaction (qRT-PCR) assays to screen female mosquito pools. The WNV duo consists of two systems targeting different genome regions: one targets the Capsid [27] and the other targets the non-coding region (3′NC) [28]. The USUV duo system consists of two assays targeting the non-structural 5 protein (NS5) [29,30] see Table 1 and Table 2. RNA extraction and presence of PCR inhibitors were monitored by spiking all the tested pools with MS2 as previously described [31]. Validation of these two assays determined that the sensitivity was 3 RNA copies and 1 RNA copy per uL for WNV and USUV, respectively.

PCR amplification was conducted using the SuperScript III Platinum One-Step qRT-PCR kit (ThermoFisher Scientific, Waltham, MA, USA) with a reaction mix of 25 µL, including 5 µL of RNA template. The reaction was carried out on an a CFX96 thermal cycler, software version 3.1 (Bio-Rad Laboratories, Hercules, CA, USA), under the following conditions: reverse transcription at 50 °C for 15 min, initial denaturation at 95 °C for 2 min, followed by 45 cycles of 95 °C for 15 s and 58 °C for 45 min.

Both assays were provided by the EVAM (European Virus Archive–Marseille) technological platform in the form of lyophilized reagents (Lyo-P&P). These assays have been produced according to the ISO13485 certification. The WNV DUO and USUV assays were accessed in the online catalog through https://www.european-virus-archive.com/evam-portal-list/field_product_type/derived-product-10150?portal_search=West%20Nile (accessed on 14 May 2025) and https://www.european-virus-archive.com/detection-kit/lyophilized-primers-and-probe-detection-usutu-virus-taqman-0 (accessed on 14 May 2025). The reagents for detecting the two viruses are routinely used by the National Reference Laboratory for Arboviruses (https://cnr-arbovirus.fr/public/ (accessed on 14 May 2025)) where they have been validated according to the COFRAC regulations. In addition, the performances of the WNV assay were assessed through the participation to the 2022 EVD-LabNet EQA.

### 2.4. Pan-Flavivirus RNA Detection

All the mosquitoes were tested by qRT-PCR with a pan-flavivirus assay using specific system targeting the Non-structural protein 5 (NS5) of the flaviviruses (Table 3) [32]. A one-step qRT-PCR assay using SYBR Green and melting curve analysis was performed by using 1× GoTaq qPCR Master Mix (Promega) with a reaction mix of 20 µL, including 4 µL of RNA template. The reaction was carried out on a thermocycler QuantStudio™ 12K Flex Real-Time PCR System (Thermo Fisher Scientific, Waltham, MA, USA), under the following conditions: reverse transcription at 50 °C for 15 min, initial denaturation at 95 °C for 10 min, followed by the PCR stages: 40 cycles of 95 °C for 10 s, 50 °C for 30 s and 75 °C for 1 min. To finish the melt curve stage includes two cycles: 60 °C for 1 min followed by 95 °C for 15 s.

## 3. Results

### 3.1. Mosquito Collection

A total of 10,617 overwintering female mosquitoes were collected in different places in northeastern France during the cold season between October 2021 to February 2024. The morphological identification of mosquitoes revealed four genera: *Culex* spp. (9360 specimens, 963 pools), *Culiseta* spp. (825 specimens, 101 pools), *Anopheles* spp. (421 specimens, 52 pools), *Aedes* spp. (11 specimens, 5 pools) (Figure S1).

### 3.2. Viral Screening

All 1121 mosquito pools tested by qRT-PCR were negative for West Nile virus, Usutu virus, and pan-Flavivirus (Table 4 and Table S1).

## 4. Discussion

This study screened 10,617 overwintering female mosquitoes across five departments in northeastern France using highly sensitive RT-PCR assays targeting independently WNV and USUV, and a broad pan-Flavivirus system. All pools tested negative, indicating no evidence of viral RNA persistence in these mosquitoes during the cold season. This observation alone does not challenge any hypothesis but rather adds to a mixed body of evidence for virus overwintering in the vector in northern Europe.

Several factors may explain the absence of WNV and USUV RNA detection in overwintering female mosquitoes. The mosquitoes collected may not be competent for the transmission of these viruses. But a study conducted by French researchers assessed the vector competence for *Culex pipiens* species present in northeastern France. The results showed that they were capable of transmitting WNV and USUV [5].

Overwintering mosquitoes enter a state of diapause, characterized by reduced metabolic activity and feeding cessation. In theory, the virus should survive as mosquitoes are still alive. Therefore, the absence of virus in mosquito pools suggest that field-collected mosquitoes were not infected.

Our results contrast with findings in other studies. In New York City, overwintering *Culex pipiens* were tested positive for WNV RNA during the winter of 2000, suggesting localized viral persistence in hibernating mosquitoes [17].

In South Moravia (Czech Republic), the overwintering was demonstrated by the detection of WNV lineage 2 in three out of 573 total pools (total mosquito sample size: 27,872) of *Culex pipiens* collected in 2017, marking the first evidence of overwintering WNV in Central Europe [33].

In the Netherlands, two studies were conducted to investigate the role of overwintering mosquitoes. In the first study, none of the 4200 mosquitoes collected during the winters of 2020 and 2021 tested positive for WNV or USUV by RT-PCR [34]. In the other study, one pool out of 504 total pools (total mosquito sample size: 4857) tested positive for an Africa 3 strain of USUV [35].

In Germany, a study provided the first evidence of WNV overwintering in mosquitoes. One pool of *Culex pipiens* out of 722 total pools (total mosquito sample size: 6101), collected in early March 2021 tested positive for WNV RNA [36]. There are many reasons that could explain the differences within each study and between these studies such as sampling locations, year of collection, mosquito species composition of captures, different overwintering capabilities of WNV and USUV. Also in these studies, the very low rates of positivity indicate that WNV and/or USUV are rare in dormant *Culex* populations. While overwintering mosquitoes likely contribute to viral persistence, their role appears limited given low detection rates. However, even sporadic cases could seed outbreaks when conditions favor vector activity. The discrepancy between our results and these studies may reflect differences in regional viral prevalence, mosquito population dynamics, or overwintering behaviors. For instance, the New York and Czech studies were conducted in regions with well-established WNV enzootic cycles and higher annual incidence rates. In contrast, northeastern France has experienced sporadic WNV and USUV activity. Avian mortality cases linked to Usutu are sporadic, but the infection is enzootic. Clinical expression, and thus mortality, is not only related to the intensity of viral circulation, but also to the immune status of the population and probably to the circulating strains [21]. Additionally, the mosquito species composition in France—dominated by *Culex pipiens* (88% of collected specimens)—differs slightly from other regions where *Culex* subspecies with distinct overwintering behaviors (e.g., *Culex pipiens pipiens pipiens* vs. *Culex pipiens molestus*) may influence viral survival.

While *Culex pipiens* in France has demonstrated laboratory competence for WNV and USUV transmission [22], field conditions may reduce this capacity. Viral replication in mosquitoes is temperature-dependent, and colder winters in northeastern France (compared to Mediterranean climates) may reduce viral replication below detectable thresholds. Furthermore, the absence of blood-fed females in our collections (all specimens were unfed) suggests that mosquitoes had not recently acquired the virus from avian reservoirs prior to hibernation. This contrasts with regions where late-season blood-feeding bridges viral transmission into the overwintering period. Laboratory experiments exposing *Culex pipiens* to WNV/USUV under diapause-like conditions would clarify whether physiological stressors limit viral survival.

The absence of viral RNA in mosquitoes raises questions about how WNV and USUV persist in France. One plausible explanation is reintroduction via migratory birds, which serve as primary reservoirs, but there is probably a local persistence of viruses. The virus could overwinter in the bird populations, including blackbirds. To verify this, it would be necessary to have access to hunted birds (since hunting takes place during autumn and winter). Controlling the sampling process would allow for prevalence estimation, and using dead birds would make it possible to test different organs by PCR. Similarly, WNV outbreaks in Europe often correlate with avian migration patterns rather than local overwintering. Additionally, non-mosquito pathways—such as persistent infection in reptiles or overwintering in ticks—remain underexplored but could contribute to viral maintenance.

While this study provides robust evidence against mosquito-mediated overwintering of WNV/USUV in northeastern France, several limitations warrant consideration. The collection focused on artificial overwintering sites (e.g., bunkers, caves), potentially overlooking natural habitats where mosquitoes might exhibit different behaviors.

The absence of viral persistence in overwintering mosquitoes suggests that seasonal WNV/USUV transmission in northeastern France is likely driven by reintroduction via migratory birds or localized enzootic cycles during warmer months. This has important implications for public health strategies: resources should focus on monitoring avian populations and active mosquito vectors during peak transmission seasons (June–November) rather than overwintering sites.

The absence of WNV and USUV RNA in overwintering female mosquitoes collected across northeastern France between 2021 and 2024 challenges the hypothesis that these vectors play a role in maintaining arboviral transmission during the cold season in this region. This study indicates that WNV and USUV could persist at low rates in overwintering female mosquitoes in northeastern France and increasing capture efforts could improve viral detections. While this reduces concerns about wintertime transmission, it reinforces the need for vigilant summer surveillance and interdisciplinary approaches to arbovirus management. Our results contrast with studies from other temperate regions, such as North America and Central Europe, where overwintering mosquitoes have been implicated in viral persistence. They underscore the complexity of arbovirus ecology and highlight the need to consider regional variations in vector biology, environmental conditions, and alternative viral maintenance mechanisms when designing surveillance and control strategies. Future research should explore the ecological drivers of viral persistence in temperate regions, particularly as climate change alters mosquito behavior and avian migration patterns.

## Figures and Tables

**Table 1:** Primers and probes included in the WNV-Duo qRT-PCR assay.

Reference	Primer/Probe	5′- > 3′ Sequence	Target	Position
Linke et al. [27]	ProC-F1	CCTGTGTGAGCTGACAAACTTAGT	Capsid	10–33
	ProC-R	GCGTTTTAG CATATTGACAGCC		132–153
	ProC-TM	6FAM-CCTGGTTTCTTAGACATCGAGATCTTCGTGC-TAMRA		89–113
Tang et al. [28]	WN10533-10552	AAGTTGAGTAGACGGTGCTG	3′ non-coding region	10,533–10,552
	WN10625-10606	AGACGGTTCTGAGGGCTTAC		10,625–10,606
	WN10560-10579	6FAM-CTCAACCCCAGGAGGACTGG-TAMRA		10,560–10,579

**Table 2:** Primers and probes included in the USUV-Duo qRT-PCR assay.

Reference	Primer/Probe	5′ > 3′ Sequence	Target	Position
Nikolay et al. [30]	UsuFP	CAAAGCTGGACAGACATCCCTTAC	Non-structural protein 5 (NS5)	10,189–10,212
	UsuRP	CGTAGATGTTTTCAGCCCACGT		10,270–10,292
	UsuP_FAM	6FAM-AAGACATATGGTGTGGAAGCCTGATAGGCA-TAMRA		10,226–10,255
Weissenböck et al. [29]	USU-Weiss-F	GCCAATGCCCTGCACTTT	Non-structural protein 5 (NS5)	9721–9739
	USU-Weiss-R	TCCCGAGGAGGGTTTCCA		9777–9795
	USU-Weiss-P	6FAM-CGATGTCCAAGGTCAGAAAAGACGTGC-TAMRA		9746–9773

**Table 3:** Primers and probes used in the pan-*flavivirus*-specific qRT-PCR assay [32].

Reference	Primer/Probe	5′ > 3′ Sequence	Target	Position
Scaramozzino et al. [32]	MAMD	AACATGATGGGRAARAGRGARAA	NS5 (non-structural protein 5)	9006–9029
	cFD2	GTGTCCCAGCCGGCGGTGTCATCAGC		9232–9258

**Table 4:** Summary of the collection, qRT-PCR assays and the results.

Collection Date	Number of Females Collected	PCR Assays	Results	Total Females Tested in qRT-PCR
October 2021–December 2021	2163 (225 pools)	WNV/USUV, pan-flavivirus	Negative	10,617 (1121 pools)
January 2022–February 2022 and November 2022	5838 (607 pools)		Negative	
February 2023 and October 2023	523 (71 pools)		Negative	
February 2024	2093 (218 pools)		Negative	

## Data Availability

Data is contained within the article or Supplementary Material: The original contributions presented in this study are included in the article/Supplementary Material. Further inquiries can be directed to the corresponding author.

## Supplementary Materials

The following supporting information can be downloaded at: https://www.mdpi.com/article/10.3390/v17091217/s1, Figure S1: Repartition of mosquito species by collection places; Table S1: Detailed table of collection dates and locations, collected species, and results.

## Abbreviations

The following abbreviations are used in this manuscript:

WNV	West Nile Virus
USUV	Usutu virus
qRT-PCR	real time Reverse transcriptase Polymerase Chain Reaction
PCR	Polymerase Chain Reaction
NS5	Non-Structural protein 5
Spp.	Species pluralis
RNA	Ribonucleic Acid
SAGIR	French national network for the epidemiological surveillance of wildlife diseases
MEM	Minimum Essential Medium
3’NC	3’ Non-Coding
EQA	External Quality Assessment
COFRAC	French national body for laboratory and certification accreditation
EVAM	European Virus Archive–Marseille
Lyo P&P	Primers and Probes Lyophilized
PBS	Phosphate-Buffered Saline

## References

[1] Kuchinsky S.C., Duggal N.K. (2024). Chapter Two—Usutu Virus, an Emerging Arbovirus with One Health Importance. Adv. Virus Res..

[2] Pealer L.N., Marfin A.A., Petersen L.R., Lanciotti R.S., Page P.L., Stramer S.L., Stobierski M.G., Signs K., Newman B., Kapoor H. (2003). Transmission of West Nile Virus through Blood Transfusion in the United States in 2002. N. Engl. J. Med..

[3] Iwamoto M., Jernigan D.B., Guasch A., Trepka M.J., Blackmore C.G., Hellinger W.C., Pham S.M., Zaki S., Lanciotti R.S., Lance-Parker S.E. (2003). Transmission of West Nile Virus from an Organ Donor to Four Transplant Recipients. N. Engl. J. Med..

[4] Hayes E.B., O’Leary D.R. (2004). West Nile Virus Infection: A Pediatric Perspective. Pediatrics.

[5] Martinet J.-P., Bohers C., Vazeille M., Ferté H., Mousson L., Mathieu B., Depaquit J., Failloux A.-B. (2023). Assessing Vector Competence of Mosquitoes from Northeastern France to West Nile Virus and Usutu Virus. PLOS Neglected Trop. Trop. Dis..

[6] Vilibic-Cavlek T., Petrovic T., Savic V., Barbic L., Tabain I., Stevanovic V., Klobucar A., Mrzljak A., Ilic M., Bogdanic M. (2020). Epidemiology of Usutu Virus: The European Scenario. Pathogens.

[7] Seroprevalence of West Nile and Usutu Viruses in Military Working Horses and Dogs, Morocco, 2012: Dog as an Alternative WNV Sentinel Species?|Epidemiology & Infection|Cambridge Core. https://www.cambridge.org/core/journals/epidemiology-and-infection/article/seroprevalence-of-west-nile-and-usutu-viruses-in-military-working-horses-and-dogs-morocco-2012-dog-as-an-alternative-wnv-sentinel-species/F1E419419D8A8C04D458474207430C8C.

[8] Maquart M., Dahmani M., Marié J.-L., Gravier P., Leparc-Goffart I., Davoust B. (2017). First Serological Evidence of West Nile Virus in Horses and Dogs from Corsica Island, France. Vector Borne Zoonotic Dis..

[9] Csank T., Drzewnioková P., Korytár Ľ., Major P., Gyuranecz M., Pistl J., Bakonyi T. (2018). A Serosurvey of Flavivirus Infection in Horses and Birds in Slovakia. Vector Borne Zoonotic Dis..

[10] Bażanów B., Jansen van Vuren P., Szymański P., Stygar D., Frącka A., Twardoń J., Kozdrowski R., Pawęska J.T. (2018). A Survey on West Nile and Usutu Viruses in Horses and Birds in Poland. Viruses.

[11] Diagne M.M., Ndione M.H.D., Di Paola N., Fall G., Bedekelabou A.P., Sembène P.M., Faye O., Zanotto P.M.d.A., Sall A.A. (2019). Usutu Virus Isolated from Rodents in Senegal. Viruses.

[12] Romeo C., Lecollinet S., Caballero J., Isla J., Luzzago C., Ferrari N., García-Bocanegra I. (2018). Are Tree Squirrels Involved in the Circulation of Flaviviruses in Italy?. Transbound. Emerg. Dis..

[13] Escribano-Romero E., Lupulović D., Merino-Ramos T., Blázquez A.-B., Lazić G., Lazić S., Saiz J.-C., Petrović T. (2015). West Nile Virus Serosurveillance in Pigs, Wild Boars, and Roe Deer in Serbia. Vet. Microbiol..

[14] Bournez L., Umhang G., Faure E., Boucher J.-M., Boué F., Jourdain E., Sarasa M., Llorente F., Jiménez-Clavero M.A., Moutailler S. (2020). Exposure of Wild Ungulates to the Usutu and Tick-Borne Encephalitis Viruses in France in 2009–2014: Evidence of Undetected Flavivirus Circulation a Decade Ago. Viruses.

[15] Cadar D., Becker N., Campos R.d.M., Börstler J., Jöst H., Schmidt-Chanasit J. (2014). Usutu Virus in Bats, Germany, 2013. Emerg. Infect. Dis..

[16] Simonin Y. (2024). Circulation of West Nile Virus and Usutu Virus in Europe: Overview and Challenges. Viruses.

[17] Nasci R.S., Savage H.M., White D.J., Miller J.R., Cropp B.C., Godsey M.S., Kerst A.J., Bennett P., Gottfried K., Lanciotti R.S. (2001). West Nile Virus in Overwintering Culex Mosquitoes, New York City, 2000. Emerg. Infect. Dis..

[18] Reisen W.K., Wheeler S.S. (2019). Overwintering of West Nile Virus in the United States. J. Med. Entomol..

[19] Baqar S., Hayes C.G., Murphy J.R., Watts D.M. (1993). Vertical Transmission of West Nile Virus by Culex and Aedes Species Mosquitoes. Am. J. Trop. Med. Hyg..

[20] Rosen L. (1988). Further Observations on the Mechanism of Vertical Transmission of Flaviviruses by *Aedes* Mosquitoes. Am. J. Trop. Med. Hyg..

[21] Bouchez-Zacria M., Calenge C., Villers A., Lecollinet S., Gonzalez G., Quintard B., Leclerc A., Baurier F., Paty M.-C., Faure É. (2025). Relevance of the synergy of surveillance and populational networks in understanding the Usutu virus outbreak within common blackbirds (*Turdus merula*) in Metropolitan France, 2018. Peer Community J..

[22] Decors A., Beck C. (2019). En France: Record de Circulation Du Virus Usutu. Faune Sauvag..

[23] Schopf F., Sadeghi B., Bergmann F., Fischer D., Rahner R., Müller K., Günther A., Globig A., Keller M., Schwehn R. (2025). Circulation of West Nile Virus and Usutu Virus in Birds in Germany, 2021 and 2022. Infect Dis..

[24] Lecollinet S., Blanchard Y., Manson C., Lowenski S., Laloy E., Quenault H., Touzain F., Lucas P., Eraud C., Bahuon C. (2016). Dual Emergence of Usutu Virus in Common Blackbirds, Eastern France, 2015. Emerg. Infect. Dis..

[25] Romiti F., Casini R., Del Lesto I., Magliano A., Ermenegildi A., Droghei S., Tofani S., Scicluna M.T., Pichler V., Augello A. (2025). Characterization of Overwintering Sites (Hibernacula) of the West Nile Vector Culex Pipiens in Central Italy. Parasit. Vectors.

[26] Kauffman E.B., Franke M.A., Kramer L.D., Colpitts T.M. (2016). Detection Protocols for West Nile Virus in Mosquitoes, Birds, and Nonhuman Mammals. West Nile Virus.

[27] Linke S., Ellerbrok H., Niedrig M., Nitsche A., Pauli G. (2007). Detection of West Nile Virus Lineages 1 and 2 by Real-Time PCR. J. Virol. Methods.

[28] Tang Y., Anne Hapip C., Liu B., Fang C.T. (2006). Highly Sensitive TaqMan RT-PCR Assay for Detection and Quantification of Both Lineages of West Nile Virus RNA. J. Clin. Virol..

[29] Weissenböck H., Bakonyi T., Rossi G., Mani P., Nowotny N. (2013). Usutu Virus, Italy, 1996. Emerg. Infect. Dis..

[30] Nikolay B., Weidmann M., Dupressoir A., Faye O., Boye C.S., Diallo M., Sall A.A. (2014). Development of a Usutu Virus Specific Real-Time Reverse Transcription PCR Assay Based on Sequenced Strains from Africa and Europe. J. Virol. Methods.

[31] Ninove L., Nougairede A., Gazin C., Thirion L., Delogu I., Zandotti C., Charrel R.N., De Lamballerie X. (2011). RNA and DNA Bacteriophages as Molecular Diagnosis Controls in Clinical Virology: A Comprehensive Study of More than 45,000 Routine PCR Tests. PLoS ONE.

[32] Scaramozzino N., Crance J.-M., Jouan A., DeBriel D.A., Stoll F., Garin D. (2001). Comparison of *Flavivirus* Universal Primer Pairs and Development of a Rapid, Highly Sensitive Heminested Reverse Transcription-PCR Assay for Detection of Flaviviruses Targeted to a Conserved Region of the NS5 Gene Sequences. J. Clin. Microbiol..

[33] Rudolf I., Betášová L., Blažejová H., Venclíková K., Straková P., Šebesta O., Mendel J., Bakonyi T., Schaffner F., Nowotny N. (2017). West Nile Virus in Overwintering Mosquitoes, Central Europe. Parasit. Vectors.

[34] Blom R., Schrama M.J.J., Spitzen J., Weller B.F.M., van der Linden A., Sikkema R.S., Koopmans M.P.G., Koenraadt C.J.M. (2022). Arbovirus Persistence in North-Western Europe: Are Mosquitoes the Only Overwintering Pathway?. One Health.

[35] Koenraadt C.J.M., Münger E., Schrama M.J.J., Spitzen J., Altundag S., Sikkema R.S., Munnink B.B.O., Koopmans M.P.G., Blom R. (2024). Overwintering of Usutu Virus in Mosquitoes, The Netherlands. Parasit. Vectors.

[36] Kampen H., Tews B.A., Werner D. (2021). First Evidence of West Nile Virus Overwintering in Mosquitoes in Germany. Viruses.

