# Supplementary figures and images for: Absence of West Nile and Usutu Virus Persistence in Overwintering Mosquitoes in Northeastern France: Insights from Cold-Season Surveillance
Source: Viruses. 2025 Sep 6;17(9):1217. doi: 10.3390/v17091217 (PMC12474421; doi:10.3390/v17091217)

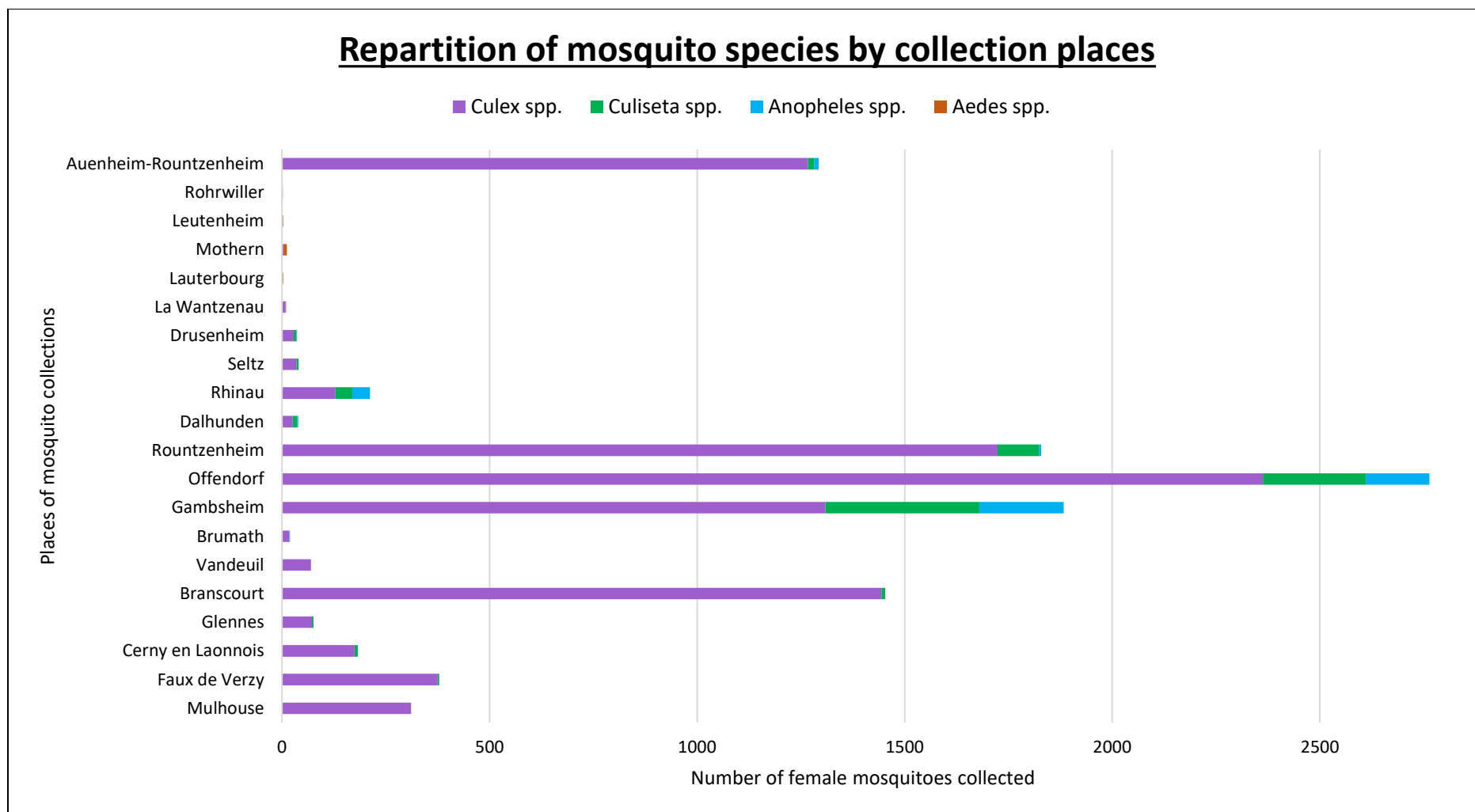

Figure S1 : Repartition of mosquito species by collection places

Supplement: Supplementary file 1 [file viruses-17-01217-s001.zip › Figure S1.pdf]
